# The prevalence of bovine mastitis-associated *Staphylococcus aureus* in China and its antimicrobial resistance rate: A meta-analysis

**DOI:** 10.3389/fvets.2022.1006676

**Published:** 2022-11-10

**Authors:** Kuan Wang, Jinlong Cha, Kai Liu, Jianming Deng, Bowen Yang, Hui Xu, Juyu Wang, Limei Zhang, Xiaolong Gu, Cuiqin Huang, Weijie Qu

**Affiliations:** ^1^College of Veterinary Medicine, Yunnan Agricultural University, Kunming, China; ^2^Yunnan Vocational and Technical College of Agriculture, Kunming, China; ^3^Department of Animal Science and Veterinary, College of Life, Longyan University, Longyan, China; ^4^Fujian Provincial Key Laboratory for Prevention and Control of Animal Infectious Diseases and Biotechnology, Longyan, China

**Keywords:** bovine mastitis, *Staphylococcus aureus*, prevalence, antimicrobial resistance, meta-analysis

## Abstract

In this study, to optimize the *Staphylococcus aureus* control program, a meta-analysis was conducted to investigate the epidemiology and antimicrobial resistance (AMR) profile of *S. aureus*-associated bovine mastitis in China from 2000 to 2020. A total of 33 publications from PubMed, Google Scholar, and China National Knowledge Infrastructure (CNKI) database were included in our research, among which nine publications included the AMR test. The pooled prevalence of *S. aureus* was 36.23%, and subgroup analysis revealed that the prevalence dropped from 2000–2010 to 2011–2020, which shows that China is on the right track. The pooled AMR rate indicate isolates were most resistant to β-lactams (50.68%), followed by quinolones (36.23%), macrolides (34.08%), sulfonamides (32.25%), tetracyclines (27.83%), aminoglycosides (26.44%), lincosamides (23.39%), and amphenicol (10.33%). Both the pooled prevalence and AMR of *S. aureus* in China are higher than those in Western countries, such as Germany, Belgium, Ireland, and the United States—countries with a long animal husbandry history and good management. Thus, there is still room to improve the treatment of *S. aureus*-associated bovine mastitis in China.

## Introduction

Bovine mastitis, as one of the most devastating diseases in dairy herds worldwide (1–3), is caused by several pathogenic bacteria, including *Staphylococcus aureus*. *Staphylococcus aureus* is one of the most prevalent pathogens worldwide and causes subclinical infections, resulting in an increased somatic cell count and intramammary infections in dairy cows (4). *S. aureus* mastitis impacts dairy farms economically because of decreased productivity, premature culling, and prolonged costly antibiotic treatments (5–7).

The resistance of *S. aureus* to antimicrobials is a growing concern, along with its wide use against the disease, although the overall resistance rates vary widely by region (8). The standard treatment regimen against bovine mastitis with antibiotics is still under debate (9). China has greatly engaged in the global action plan on antimicrobial resistance (AMR) control (10). The National Action Plan to Combat Animal Origin Antimicrobial Resistance (2017–2020) (Beijing: China Ministry of Agriculture and Rural Affairs, 2017) is one of the national protocols to standardize veterinary medications in combination with strict biosecurity measures and prudent use of antimicrobials to alleviate the pressure of resistant pathogen transmission. Significant progress has been made against the AMR by prohibiting certain antibiotics (Announcement No. 194 of the Ministry of Agriculture and Rural Affairs of the People's Republic of China), for instance, officially prohibition of the use of three veterinary drugs, namely, olaquindox [“Chinese Veterinary Pharmacopeia” (2005 Edition)], clenbuterol (Notice of the General Office of the Ministry of Agriculture and Rural Affairs on Launching the Special Rectification Action for “Clenbuterol”), and salbutamol [State Pharmacopeia Commission. 2010 Pharmacopeia of the People's Republic of China (Part 2)] in food animals to ensure the quality and safety of animal products and maintain public health and ecological safety.

The prevalence and AMR rate of *S. aureus-*related bovine mastitis in different regions of China during 2000–2020 were estimated using meta-analysis (11), an innovative tool, by analyzing the findings of published studies. Pooled prevalence and AMR rate, as well as subgroup analysis, from different aspects were conducted.

The purpose of this study was to understand the epidemiology and AMR profiles of *Streptococcus* spp. using meta-analysis to optimize *Streptococcus* spp. control programs.

## Materials and methods

### Literature search

**Figure 1:**
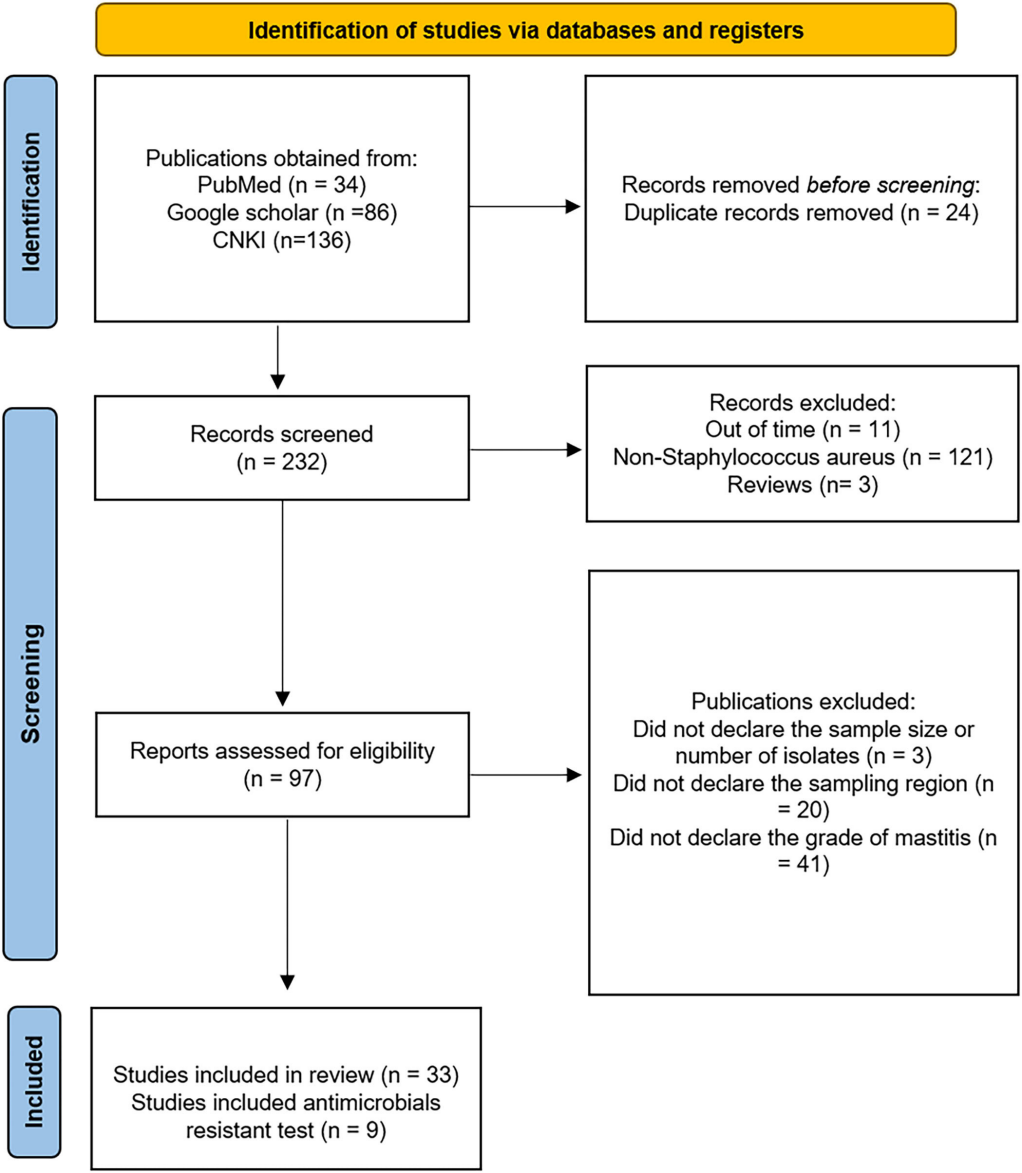
Identification of studies *via* databases and registers.

Literature retrieval steps and results are illustrated in Figure 1. A comprehensive and systematic literature search was conducted to identify studies on *S. aureus-*related bovine mastitis, utilizing PubMed (www.pubmed.gov), Google Scholar (https://scholar.google.com), and China National Knowledge Infrastructure (CNKI) database (https://www.cnki.net/). “Bovine mastitis AND bacteria” were used as key words fothe search of publications in English and Chinese between 2000 and 2020.

### Selection of published studies

The PRISMA reporting standard was adopted in this study, as previously reported (12–14). Articles were excluded if (a) they were duplicate records; (b) they went off-topic and had small sample size (< 3); (c) the study did not involve bacterial identification; (d) the study samples contained non-mastitis diseases; (e) the study involved ambiguous sample size or bacterial isolate quantity, and (f) the study was conducted out of the defined period (before 2000 and after 2020). Microsoft Excel was used to manage the references (Table 1).

**Table 1:** Information of studies included in our study.

**Author**	**Year**	**Sample**	**Identification assay**	**Isolates**	**Grade^a^**	**Region^b^**	**AMR method^c^**
Meng Dan	2019	186	16S	98	C	N	
Weijie Jin	2020	544	16S	168	C	S	K–B
Lili Zhang	2016	200	Other	58	C	S	K–B
Qiang Ren	2019	84	16S	65	S	N	K–B
Feng Li Yang	2014	67	Other	12	C	S	
Chenchen Shen	2017	28	Other	18	C	S	K–B
Mingxu Zhou	2019	50	16S	5	S	S	K–B
Huiyun Zhao	2020	110	16S	15	C	N	K–B
Weize Gan	2020	812	16S	216	S	N	K–B
Haiyan Wu	2019	50	Other	18	C	N	K–B
Lijun Wu	2019	165	16S	43	S	S	
Wei Liu	2006	60	Other	43	C	N	
Lei Liu	2009	92	Other	58	S	N	
Yu Li	2011	16	Other	12	C	N	
Jin Li	2014	58	Other	53	C	N	
Lin Wang	2015	100	Other	15	C	N	
Hongwei He	2015	14	Other	12	C	N	
Xiujuan Ye	2004	44	Other	30	C	S	
Jianbiao Lu	2006	63	Other	23	C	N	
Ying Liu	2008	90	Other	23	C	N	
Guiying Wang	2008	115	Other	12	C	N	
Yongxin Yang	2009	86	Other	42	S	S	
Lulu Qin	2009	30	Other	4	C	S	
Guixian Zhang	2010	34	Other	6	C	N	
Fu Cong	2007	304	Other	91	S	N	
Long Ma	2009	44	Other	29	C	N	
Zhuming Zhang	2009	9	Other	5	C	N	
Xiaodong Kang^a^	2014	94	Other	7	C	N	
Xiaodong Kang^b^	2014	164	Other	11	C	N	
Jie Lin	2015	15	16S	10	C	N	
Xinpu Li	2015	302	16S	18	C	N	
Qiuyun Zhao	2016	48	Other	10	C	N	K–B
Liming Chen	2004	23	Other	12	C	S	
Yan Liu	2012	114	Other	63	C	S	
Total		4,215	–	1,305	–	–	–

^a^C, clinical bovine mastitis; S, subclinical bovine mastitis. ^b^S, South China; N, North China. ^c^K–B, disk diffusion test; –, publication did not include the AMR test.

### Data extraction and statistical analysis

Designed forms were used to extract data from the selected publications, and the data included author, year of publication, province, sample size, number of *S. aureus* isolates, degree of mastitis (as per the Laboratory Handbook on Bovine Mastitis, National Mastitis Council), identification method, number of resistant isolates, and laboratory procedure. The methodological quality of each study was independently reviewed by two reviewers based on pre-specified study quality indicators adapted from the Downs and Black checklist.

The number of *S. aureus*, antimicrobial-resistant isolates, and mastitis milk samples of the extracted data were calculated for their proportion in articles. Resistance was considered a dichotomous outcome. The prevalence and AMR rate were separately meta-analyzed by using the “meta” and “metafor” packages in R (version 4.0.5).

The prevalence of *S. aureus* was pooled using the random effects model. Subgroup meta-analyses were conducted on isolation time and region, and mastitis grade to illustrate the heterogeneity between the studies.

The AMR profile was analyzed by groups: β-lactams, quinolones, aminoglycosides, tetracyclines, lincosamides, sulfonamides, macrolides, and amphenicol. The publication bias test was performed by using the Egger test, and a funnel plot was created.

## Results

### Inclusion of publications

A total of 34, 86, and 136 articles were obtained from PubMed, Google Scholar, and CNKI, respectively, among which the following were excluded: 24 publications were duplicates, 11 were published out of the defined period, 121 did not involving *S. aureus*, three were reviews, three did not provide information of sample size or the number of bacterial isolates, 20 did not provide data on the sampling region, and 41 did not provide grade of mastitis. As a result, 33 publications including 4,215 samples and 1,305 isolates were selected for subsequent analysis, of which nine were included for the AMR test (Figure 1, Table 1).

### Prevalence of *S. aureus*

**Figure 2:**
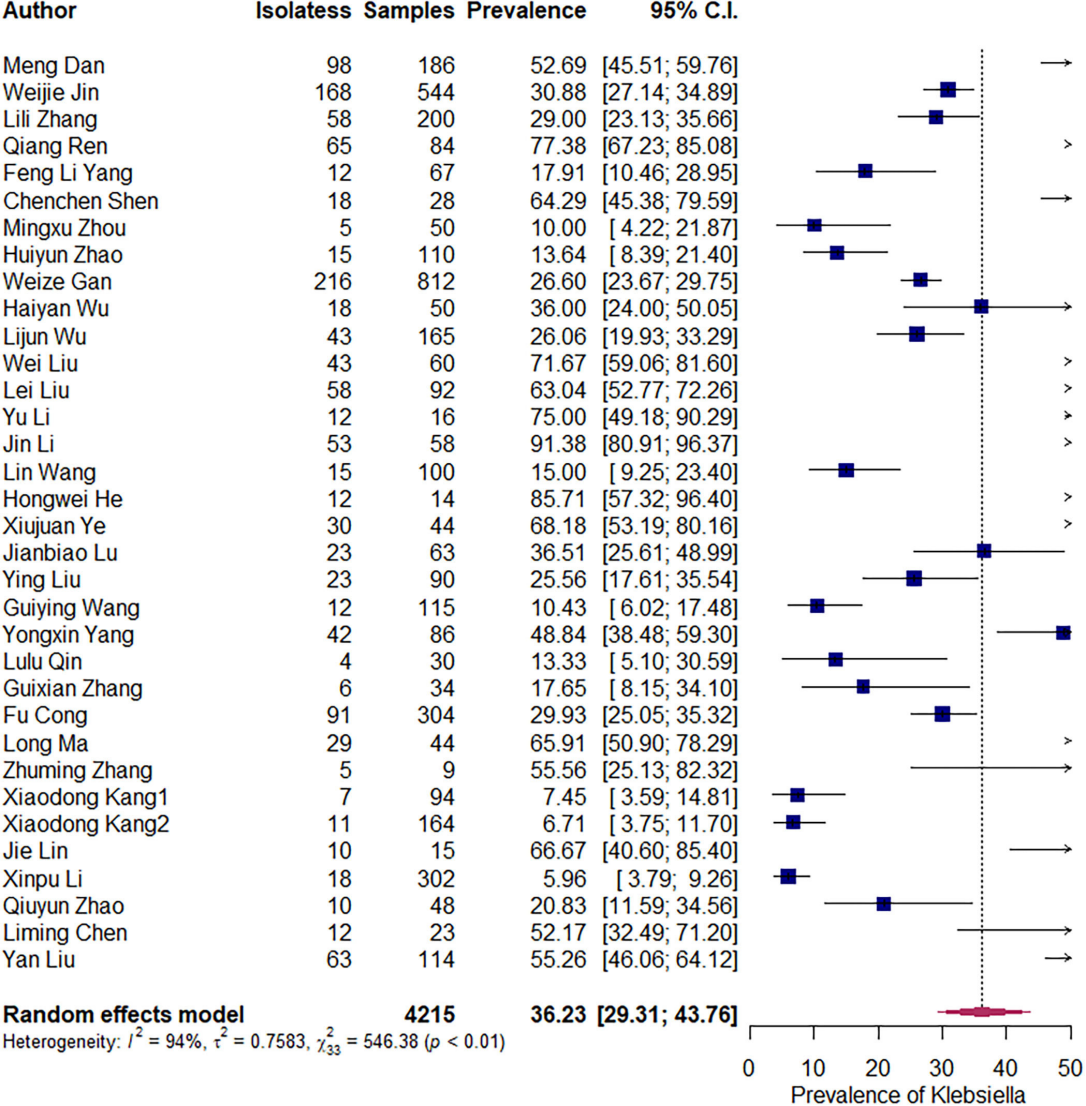
Overall prevalence of *Staphylococcus aureus*.

The pooled prevalence of *S. aureus* is 36.23% [95% confidence interval (CI): 29.31–43.76%]. An evident heterogeneity was observed (*I*^2^ = 94%, *t*^2^ = 0.7583, *P* < 0.01). Therefore, subgroup analysis was conducted to explore the sources of heterogeneity (Figure 2).

### Subgroup analysis

**Figure 3:**
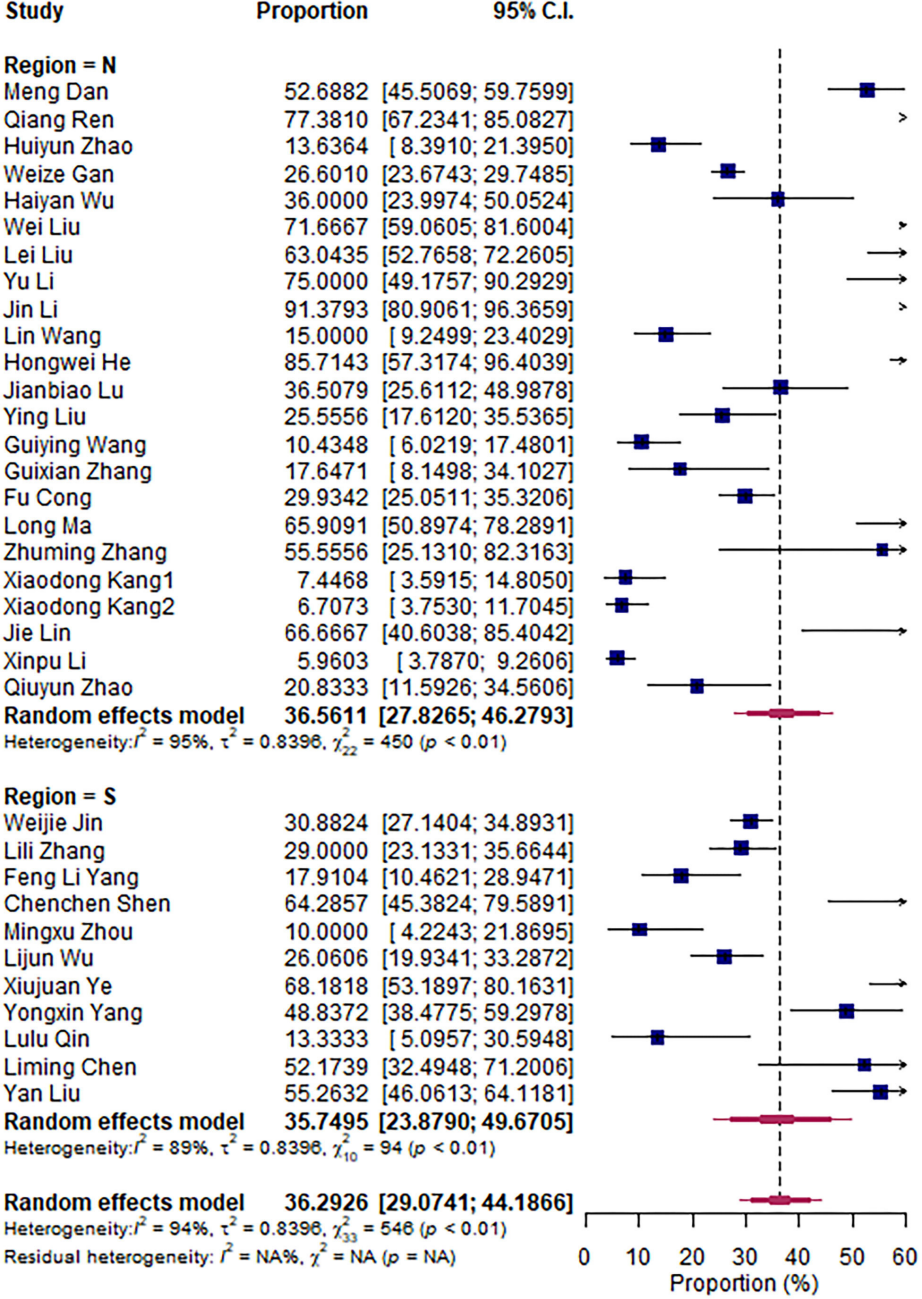
Prevalence subgroup by region.

**Figure 4:**
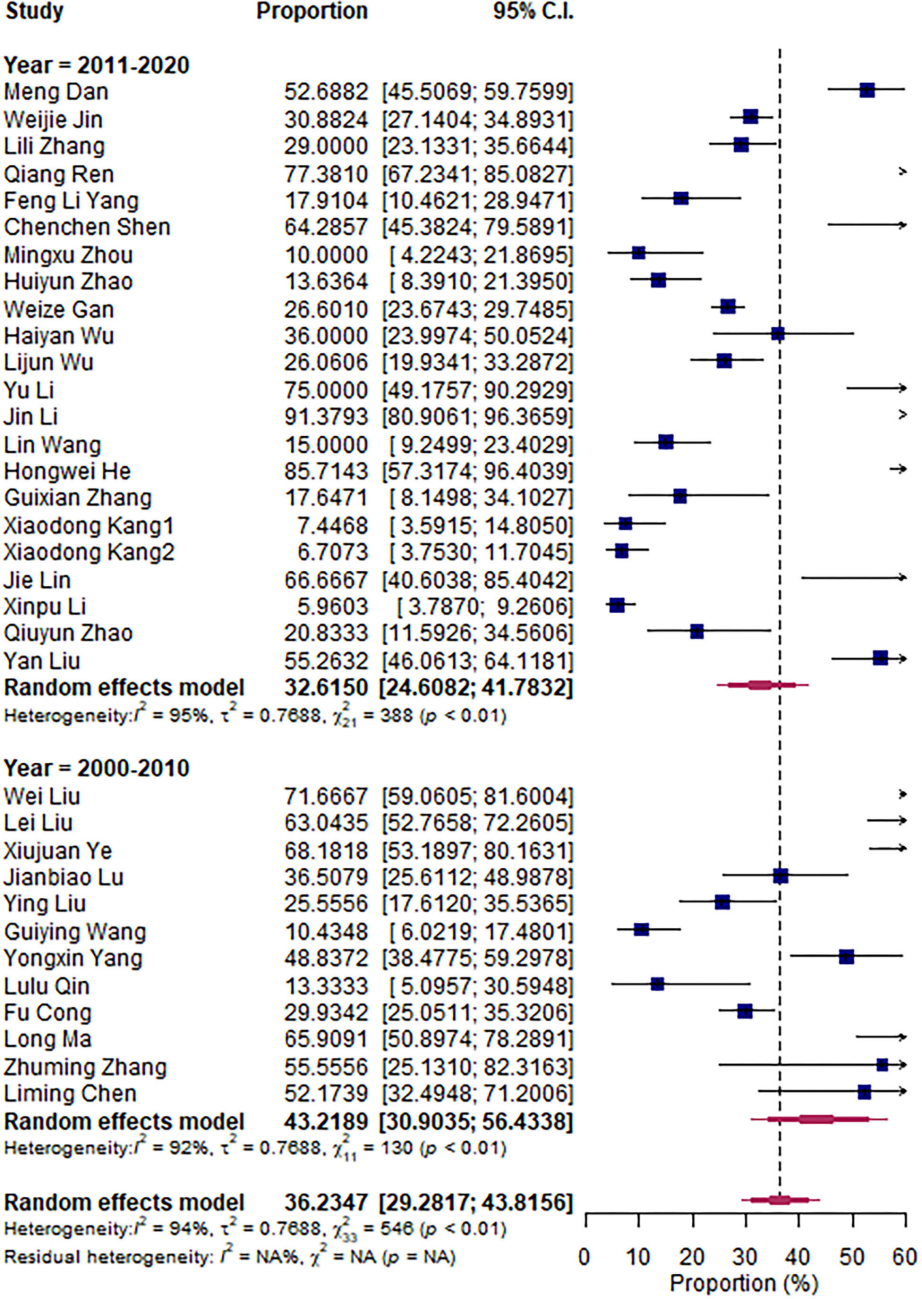
Overall prevalence subgroup by year.

**Figure 5:**
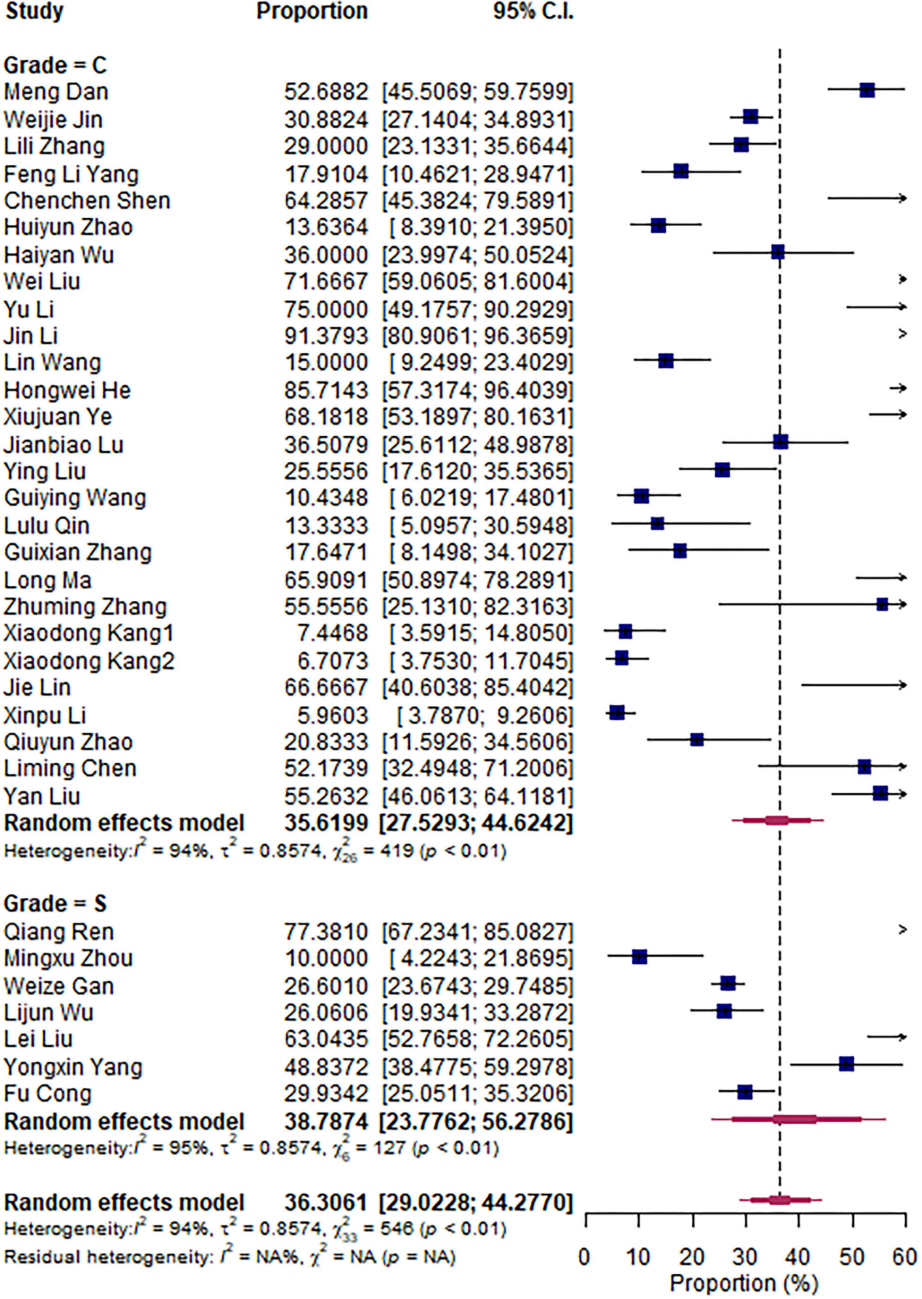
Overall prevalence subgroup by grade.

The research articles were divided into subgroups based on research period (2000–2010 vs. 2011–2020), sampling sites (North vs. South China), and mastitis grade (clinical vs. subclinical mastitis). The pooled subgroup prevalence of *S. aureus* was 36.56% and 35.75% in North and South China, respectively (Figure 3); 43.22 and 32.62% for the 2000–2010 period and the 2011–2020 period, respectively (Figure 4); and 35.62% and 38.79% in clinical and subclinical mastitis, respectively (Figure 5).

According to the aforementioned meta-analysis results, we speculate that the difference in prevalence between South China and North China and the difference between periods 2000–2010 and 2011–2020 may be related to the difference in climate between North and South China and the increased emphasis on *Streptococcus agalactiae*, which is related to factors such as the improvement of biological prevention and control.

### Antimicrobial resistance rate of *S. aureus*

**Figure 6:**
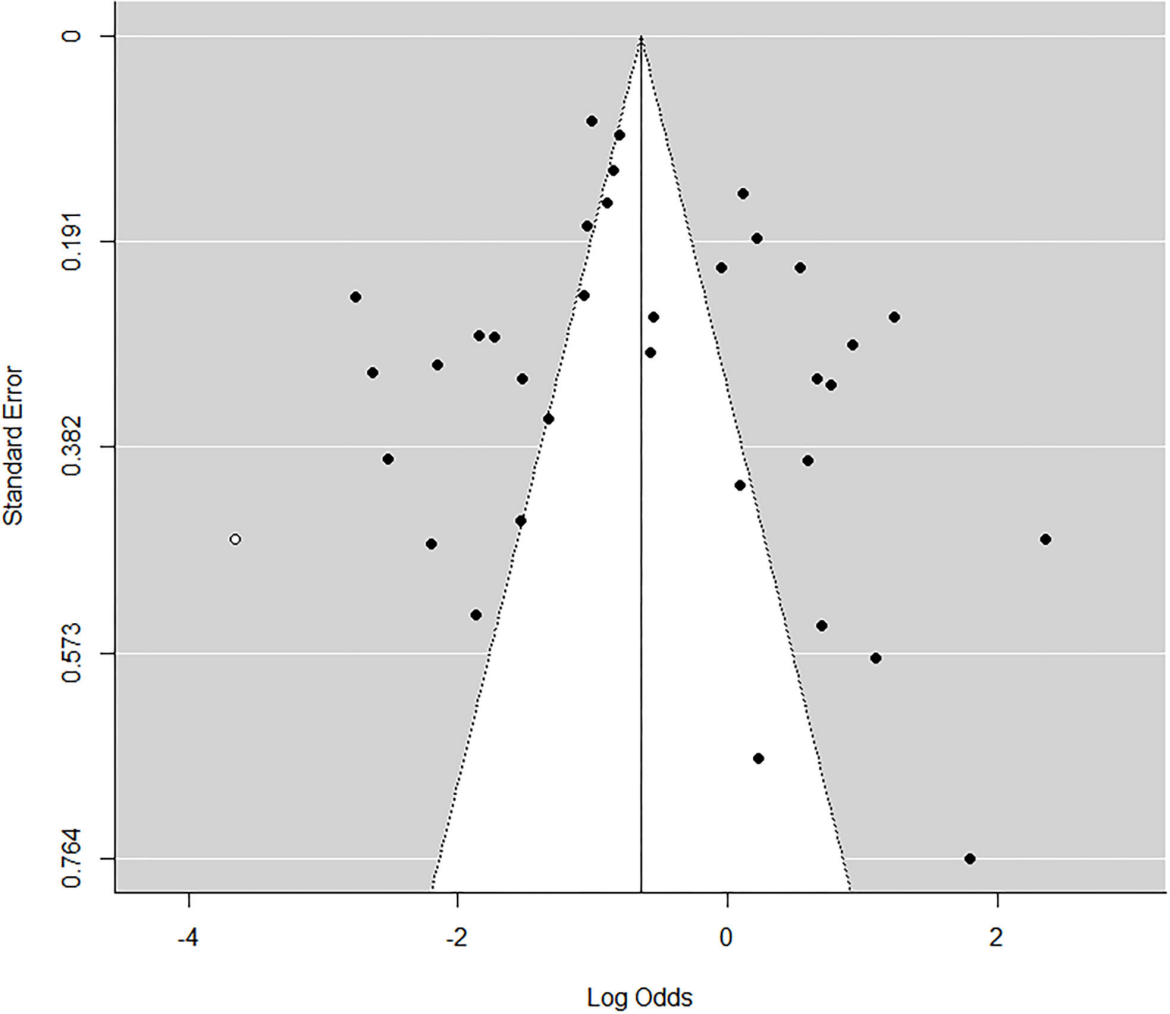
Antimicrobial resistance rate of *Staphylococcus aureus*.

The pooled antimicrobial resistant rate revealed that *S. aureus* was most resistant to β-lactams, 50.68% (95% CI: 42.55–58.77%); followed by quinolones, 36.23% (95% CI: 28.45–44.79%); macrolides, 34.08% (95% CI: 26.89–42.08%); sulfonamides, 32.25% (95% CI: 20.81–46.30%); tetracyclines, 27.83% (95% CI: 21.29–35.46%); aminoglycosides, 26.44% (95% CI: 19.33–35.02%); lincosamides, 23.39% (95% CI: 16.70–31.74%); and amphenicol, 10.33% (95% CI: 6.07–17.18%) (Figure 6).

### Publication bias of the prevalence and AMR rate of *S. aureus*

**Figure 7:**
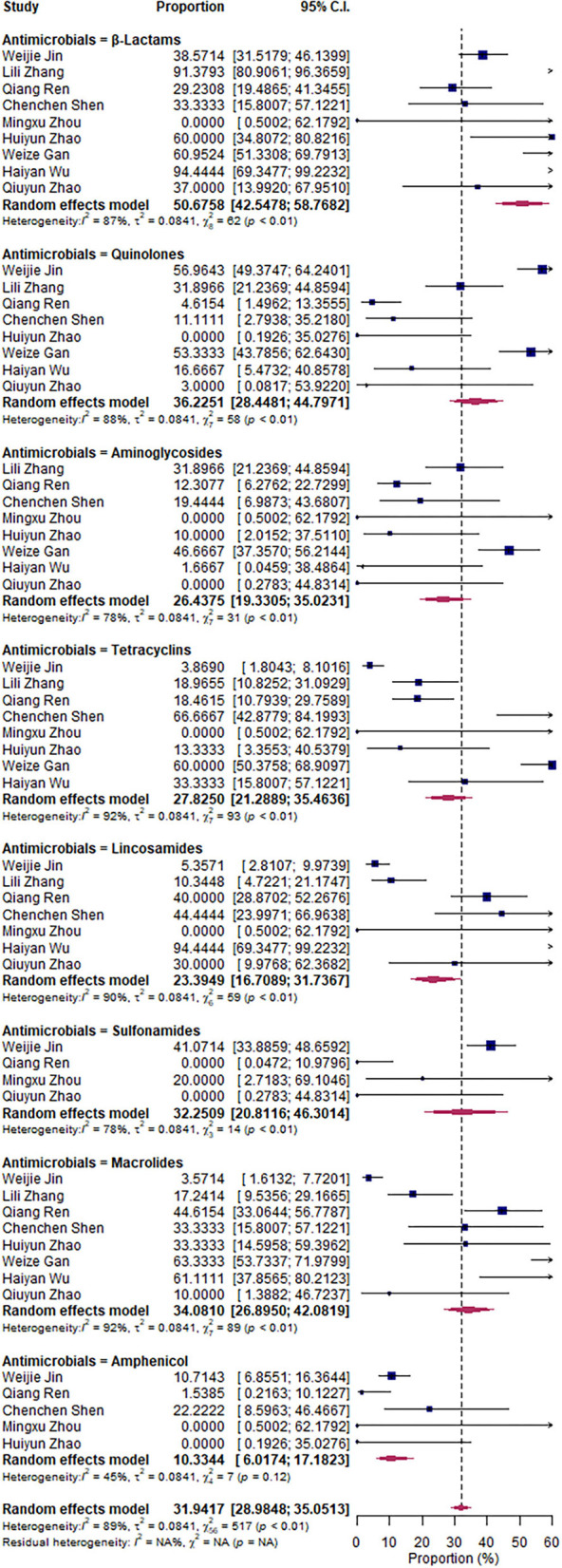
Funnel plot of prevalence bias of *Staphylococcus aureus*.

**Figure 8:**
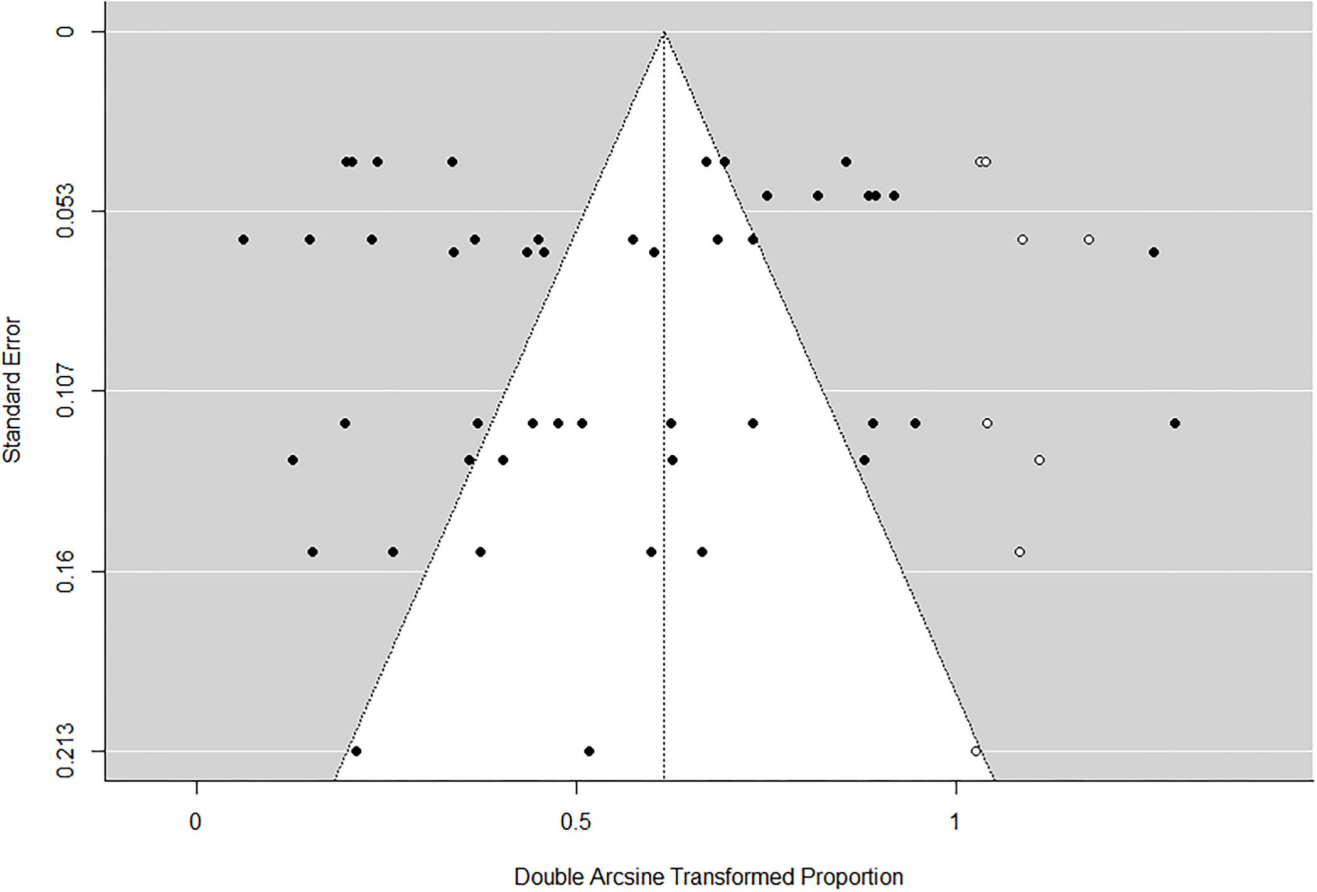
Publication bias of antimicrobial resistant rate of *Staphylococcus aureus*.

As shown by the funnel plot (Figures 7, 8), the studies exhibited an even distribution around the mean effect size, which suggested the publication bias is negligible.

## Discussion

Bovine mastitis is a disease of dairy cows worldwide (2, 15). *S. aureus* is one of the main pathogens causing the disease (16, 17) and is also the third largest foodborne pathogen in the world, posing a huge threat to animal husbandry and human public health (4), causing economic losses up to €300 per cow per year (18, 19), and thus fueling the increase of clinical, subclinical, and recurrent cow mastitis (6, 20). It is essential to understand the prevalence and AMR rate of bovine mastitis-related *S. aureus* to improve therapeutic interventions and prevention strategies.

The pooled prevalence of *S. aureus* in China (36.23%) is lower than that in the United States (46.6 ~ 62.4%, 118 of 189 herds) (21), Hungary (70%) (22), Northern Greece (40%) (23), and northern Ethiopia (41.7%) (24), but is higher than that in Denmark (34%) (25), Germany (7.3 ~ 11.5%) (26), Belgium (7.6%) (26), Iran (25%) (27), Japan (28.2%) (28), Nepal (15.2%) (29), and Korea (5.6%) (30). The difference in prevalence between China and the United States may be due to the fact that the scale of the United States is generally larger than that in China, and the farms covered in our study are only partial farms, and there may also be some high prevalence undetected. Apart from that, Patel et al. (21) suggested that caution should be exercised when generalizing the findings of smaller herds, so prevalence will still vary considerably between China and other developed countries. The fact that China is the third largest milk producer in the world may be the reason for the higher prevalence. It could be concluded that further measures need to be taken against bacterial resistance and to improve related managements in farms (2, 31, 32).

Song et al. (32) and Gao et al. (31) suggested that the higher prevalence in North China may due to colder winters and lack of heat, reluctance to keep up with the rapid development of farming technology and so forth. Another important reason may lie in the fact that the dairy industry is much more developed in North China, where the main dairy zone and the large-scale farms are located. However, in the study by Gao et al. (31), some samples were stored at 4°C, instead of a freezer; repeated freeze–thaw of the sample reduces the culture sensitivity of the bacteria. In addition, as mentioned in the study of Gao et al. we were unable to interpret the findings because of the lack of management details of the studied herds (31).

In our analysis, the prevalence of subclinical mastitis caused by *S. aureus* is higher than that of clinical mastitis, which is inconsistent with the fact that clinical mastitis is more common than subclinical mastitis (32) but is consistent with the fact that a higher incidence of subclinical mastitis is predominant (33). Meanwhile, the incidence of the clinical type of bacteriologic bovine mastitis was roughly 20 ~ 22% in Canada (34), and that of subclinical mastitis was about 20.8 ~ 23.3% in the United States (35). Moreover, considering the different research angles and the limitations of the sample size used in the analysis, the difference is not surprising (34, 35). The specific prevalence of clinical and subclinical mastitis in China requires further meticulous studies to draw more accurate conclusions.

The lower prevalence in the recent decade of 2011–2020 than the decade of 2000–2010 (32.62 vs. 43.22%) might imply the decline in the prevalence due to the rapid technology development against *S. aureus* and biosecurity measures undertaken by the farms. This may be a good sign that *S. aureus* could be more effectively controlled in future along with the development of more advanced technology and increased attention paid to the industry (36). Mammary gland health is further complicated by differences in farm management systems, farm sizes, cow cleanliness, and housing styles across countries and regions (37).

In our study, *S. aureus* is the most resistant bacterium to β-lactams (50.68%). It was shown in the study by Perovic that *S. aureus* may have an acquired gene that makes it resistant to methicillin and to all other β-lactam antibiotics (28, 38). In addition, penicillin belongs to the β-lactam class of drugs, the drug has been used for long-term and repeated administration in cattle, for example, for the treatment of diarrhea and other diseases, which may result in increased resistance to its use in the treatment of clinical mastitis (39); hence, β-lactams might be the most resistant antibiotic against *S. aureus*. This result is supported by studies conducted in Iran (27) and Brazil (40), both showing that *S. aureus* is highly resistant to β-lactam antibiotics compared with other antibiotics. In India, resistance to oxacillin (a penicillin drug) can reach 20.5% (41). In Japan, the resistance to ampicillin can reach 76.1%~89.7% (28). In Nepal, *S. aureus* isolates were totally (100%) resistant to ampicillin, 75.9% to cefazolin, and 48.3% to tetracycline (29).

China and other countries follow different practices regarding the use of antibiotics (42–44). However, rational evaluation, drug screening, and cautious and responsible use are meaningful to all countries to gradually reduce the use of antibiotics in veterinary practice in future.

## Conclusion

The pooled prevalence of *S. aureus* was 36.23%, and subgroup analysis revealed that the prevalence was higher in North China in 2000–2010 and in subclinical bovine mastitis cases. Pooled AMR rates revealed *S. aureus* is highly resistant to β-lactams and quinolones; therefore, caution should be taken against treatments involving these two types of antibiotics for bovine mastitis.

## Data availability statement

The original contributions presented in the study are included in the article/supplementary material, further inquiries can be directed to the corresponding author.

## Author contributions

KW, JC, and KL contributed to conception and design of the study. KL organized the database. KW performed the statistical analysis and wrote the first draft of the manuscript. KL, JD, BY, HX, and JW wrote sections of the manuscript. LZ, CH, and XG performed the literatures research and review. WQ critically reviewed and revised the manuscript. All authors read and approved the final version.

## Funding

This study was funded by the National Natural Science Foundation of China (Grant No. 31660730), Open Fund Project of Longyan University and Fujian Provincial Key Laboratory for Prevention and Control of Animal Infectious Diseases and Biotechnology (ZDSYS2022003), Yunnan Expert Workstation (Grant No. 202005AF150041), and Veterinary Public Health Innovation Team of Yunnan Province (Grant No. 202105AE160014).

## Conflict of interest

The authors declare that the research was conducted in the absence of any commercial or financial relationships that could be construed as a potential conflict of interest.

## Publisher's note

All claims expressed in this article are solely those of the authors and do not necessarily represent those of their affiliated organizations, or those of the publisher, the editors and the reviewers. Any product that may be evaluated in this article, or claim that may be made by its manufacturer, is not guaranteed or endorsed by the publisher.
